# Resposta Cardíaca ao Estresse: Influência da Vortioxetina

**DOI:** 10.36660/abc.20250105

**Published:** 2025-04-07

**Authors:** Rodrigo Guimarães Vieira de Carvalho, Gustavo Augusto Ferreira Mota, Milene Vitória Sampaio Sobral, Ana Paula Coelho Figueira Freire, Francis Lopes Pacagnelli

**Affiliations:** 1 Universidade do Oeste Paulista Presidente Prudente SP Brasil Universidade do Oeste Paulista, Presidente Prudente, SP – Brasil; 2 Universidade Estadual Paulista Faculdade de Medicina de Botucatu Departamento de Clínica Médica São Paulo SP Brasil Departamento de Clínica Médica – Faculdade de Medicina de Botucatu – Universidade Estadual Paulista (UNESP), São Paulo, SP – Brasil; 3 Central Washington University Ellensburg WA EUA Central Washington University, Ellensburg WA – EUA

**Keywords:** Antidepressivos, Coração, Estresse Psicológico, Vortioxetina

O estresse psicológico é comum e desempenha um papel significativo na adaptação aos desafios ambientais. No entanto, também tem efeitos negativos para a saúde, potencialmente desencadeando transtornos psiquiátricos, como ansiedade e depressão.^1^ As respostas fisiológicas ao estresse desempenham um papel crucial no desenvolvimento de doenças cardiovasculares, pois as alterações hemodinâmicas, vasculares e imunológicas induzidas pelo estresse estão particularmente envolvidas neste processo.^2^ O estresse ativa regiões do cérebro que, por meio do sistema nervoso simpático ou hormônios, afetam a função cardíaca. O estresse leve imprevisível crônico (ECMI) aumenta a corticosterona sérica, agravando os perfis lipídicos e acelerando a aterosclerose em roedores.^3^ O estresse crônico ativa o eixo hipotálamo-hipófise-adrenal, aumentando os glicocorticoides e as espécies reativas de oxigênio, contribuindo para os distúrbios cardiovasculares.^4^

Os modelos ECMI simulam depressão em animais, evidenciada pela anedonia, que é reversível com antidepressivos.^5^ Este modelo animal foi validado e é amplamente utilizado; no entanto, melhorias em estudos que utilizam este modelo podem ser implementadas. Recomenda-se estratificar os animais no grupo ECMI em coortes "resilientes" e "suscetíveis", usar protocolos mais refinados no teste de preferência por sacarose para minimizar artefatos fisiológicos e físicos, avaliar sistematicamente os efeitos não específicos do ECMI e implementar ajustes em testes comportamentais.^6^ Os pesquisadores observaram que ratos expostos ao ECMI exibiram alterações cardíacas e comportamentais, aumentando o risco de arritmias e infarto.^7^

Embora os antidepressivos possam afetar o coração, a vortioxetina (VOR) surge como uma opção promissora com efeitos neuroprotetores e cardiovasculares positivos.^8^ A VOR é um antidepressivo indicado para depressão maior, particularmente quando acompanhada de comprometimento cognitivo. A VOR atua multimodalmente, funcionando como um antagonista dos receptores de serotonina 5-HT3, 5-HT7 e 5-HT1D, um agonista parcial de 5-HT1B, um agonista total de 5-HT1A e um inibidor de transportadores de serotonina.^8^ Embora o impacto do estresse nas doenças cardiovasculares seja amplamente reconhecido, seus efeitos diretos no miocárdio e o papel dos agentes farmacológicos neste contexto permanecem pouco explorados.

Em publicação recente nos Arquivos Brasileiros de Cardiologia, Ozmen et al.^9^ investigaram os potenciais efeitos do VOR sobre aspectos histopatológicos e imuno-histoquímicos em modelo animal submetido a ECMI. O uso do VOR melhorou alterações histológicas no ventrículo esquerdo, como hiperemia, edema, degeneração gordurosa, degeneração vacuolar, hipereosinofilia, aumento de células mononucleares e hemorragia miocárdica. Também foi demonstrado um potencial efeito cardioprotetor contra apoptose (redução da caspase-3), inflamação (diminuição do NF-κB e aumento da IL-10) e lesão cardíaca (redução da troponina).

Outras avaliações miocárdicas após a administração de VOR devem ser investigadas para confirmar seu efeito cardioprotetor. A ativação do sistema renina-angiotensina-aldosterona, particularmente a angiotensina 2 e o receptor acoplado ATR-1, foram identificados na hipertrofia cardíaca relacionada ao ECMI em associação com a ativação do sistema nervoso simpático.^10^ Portanto, estudos futuros são necessários para confirmar o papel do VOR em outros aspectos cardíacos histológicos induzidos pelo estresse.
