# Supplementary material for: Resposta Cardíaca ao Estresse: Influência da Vortioxetina
Source: Arq Bras Cardiol. 2025 Apr 7;122(2):e20250105. [Article in Portuguese] doi: 10.36660/abc.20250105 (PMC12040386; doi:10.36660/abc.20250105)
Supplement: Translation

# Translation: Cardiac Response to Stress: Influence of Vortioxetine

**DOI:** 10.36660/abc.20250105i

**Authors:** Rodrigo Guimarães Vieira de Carvalho, Gustavo Augusto Ferreira Mota, Milene Vitória Sampaio Sobral, Ana Paula Coelho Figueira Freire, Francis Lopes Pacagnelli

**Affiliations:** 1 Presidente Prudente SP Brazil Universidade do Oeste Paulista, Presidente Prudente, SP – Brazil; 2 São Paulo SP Brazil Departamento de Clínica Médica – Faculdade de Medicina de Botucatu – Universidade Estadual Paulista (UNESP), São Paulo, SP – Brazil; 3 Ellensburg WA USA Central Washington University, Ellensburg WA – USA

**Keywords:** Antidepressive Agents, Heart, Psychological Stress, Vortioxetine

Psychological stress is common and plays a significant role in adapting to environmental challenges. However, it also has negative health effects, potentially triggering psychiatric disorders such as anxiety and depression.^1^Physiological responses to stress play a crucial role in the development of cardiovascular diseases, as hemodynamic, vascular, and immunological changes induced by stress are particularly involved in this process.^2^Stress activates brain regions that, through the sympathetic nervous system or hormones, affect heart function. Chronic unpredictable mild stress (CUMS) increases serum corticosterone, aggravating lipid profiles and accelerating atherosclerosis in rodents.^3^Chronic stress activates the hypothalamic-pituitary-adrenal axis, increasing glucocorticoids and reactive oxygen species, contributing to cardiovascular disorders.^4^

CUMS models simulate depression in animals, as evidenced by anhedonia, which is reversible with antidepressants.^5^This animal model has been validated and is widely used; however, improvements in studies utilizing this model can be implemented. It is recommended to stratify animals in the CUMS group into "resilient" and "susceptible" cohorts, use more refined protocols in the sucrose preference test to minimize physiological and physical artifacts, systematically evaluation of the nonspecific effects of CUMS, and implement adjustments in behavioral tests.^6^Researchers observed that rats exposed to CUMS exhibited both cardiac and behavioral changes, increasing the risk of arrhythmias and infarction.^7^

Although antidepressants can affect the heart, vortioxetine (VOR) emerges as a promising option with positive neuroprotective and cardiovascular effects.^8^VOR is an antidepressant indicated for major depression, particularly when accompanied by cognitive impairment. VOR acts multimodally, functioning as an antagonist of serotonin receptors 5-HT3, 5-HT7, and 5-HT1D, a partial agonist of 5-HT1B, a full agonist of 5-HT1A, and an inhibitor of serotonin transporters.^8^While the impact of stress on cardiovascular diseases is widely recognized, its direct effects on the myocardium and the role of pharmacological agents in this context remain underexplored.

In a recent publication in the *Arquivos Brasileiros de Cardiologia* , Ozmen et al.^9^investigated the potential effects of VOR on histopathological and immunohistochemical aspects in an animal model undergoing CUMS. The use of VOR improved histological alterations in the left ventricle, such as hyperemia, edema, fatty degeneration, vacuolar degeneration, hypereosinophilia, increased mononuclear cells, and myocardial hemorrhage. A potential cardioprotective effect against apoptosis (reduction of caspase-3), inflammation (decrease in NF-κB and increase in IL-10), and cardiac injury (reduction of troponin) was also demonstrated.

Further myocardial assessments following VOR administration should be investigated to confirm its cardioprotective effect. The activation of the renin-angiotensin-aldosterone system, particularly Angiotensin 2 and the ATR-1 coupled receptor, were identified in CUMS-related cardiac hypertrophy in association with sympathetic nervous system activation.^10^Therefore, future studies are necessary to confirm the role of VOR in other histological cardiac aspects induced by stress.
